# Perspectives of clinicians and patients on community-based maintenance care for adults with obesity

**DOI:** 10.1093/eurpub/ckac131.304

**Published:** 2022-10-25

**Authors:** K McBride, G Alsultany, J Termaat, K Williams

**Affiliations:** 1 School of Medicine, Western Sydney University, Penrith, Australia; 2 Nepean Family Metabolic Health Service, Nepean Local Health District, Penrith, Australia

## Abstract

**Background:**

Tertiary metabolic health services are in high demand as people with severe obesity increase. Once predetermined health goals have been achieved patients must transition to community-based care to urgently free up capacity in tertiary services. Maintenance of successful outcomes achieved via tertiary services is therefore important to limit rates of relapse back to these services.

**Methods:**

This qualitative project explored community-based care needs to help individuals living with obesity maintain health gains. An interview schedule guided one-on-one interviews with patients and staff from metabolic clinics in Sydney, Australia.

**Results:**

We interviewed 22 patients and 13 clinicians. A lack of appropriate and consistent clinical support in the community was identified by patients and clinicians. Most clinicians agreed primary care was key to successful maintenance care. Lack of primary care understanding of appropriate management and support for patients with obesity, lack of bariatric equipment and limited funding for allied health were all seen barriers to appropriate support beyond their clinics. Patients were highly reluctant to transition from tertiary clinics and reluctant to engage with community-based care due to experience of limited clinical/social support and bariatric equipment, demeaning clinical interactions, lack of care coordination and being stigmatised. Support groups outside of the clinic were also identified important in mitigating social isolation and stigma. Both patients and clinicians felt support groups have potential to provide important supplementary help to individuals with obesity outside tertiary settings.

**Conclusions:**

Currently, individuals aiming to maintain their weight are likely to struggle in the context of existing community care provisions. Integrated, community-based and affordable models of care are needed now to allow tertiary metabolic services discharge their patients safely.

**Key messages:**

• Tertiary obesity services are at capacity.

• Subsequent community care for people wth obesity needs to be mote appropriate tp promote weight maintenance.

